# Seroprevalence of Unidentified SARS-CoV-2 Infection in Hong Kong During 3 Pandemic Waves

**DOI:** 10.1001/jamanetworkopen.2021.32923

**Published:** 2021-11-15

**Authors:** Siaw S. Boon, Martin C. S. Wong, Rita W. Y. Ng, Danny T. M. Leung, Zigui Chen, Christopher K. C. Lai, Wendy C. S. Ho, Junjie Huang, Barry K. C. Wong, Kitty S. C. Fung, Paul K. S. Chan

**Affiliations:** 1 Department of Microbiology, Faculty of Medicine, Prince of Wales Hospital, The Chinese University of Hong Kong, Hong Kong, China; 2 Jockey Club School of Public Health and Primary Care, Faculty of Medicine, The Chinese University of Hong Kong, Hong Kong, China; 3 Department of Chemical Pathology, Faculty of Medicine, The Chinese University of Hong Kong, Hong Kong, China; 4 Department of Pathology, United Christian Hospital, Hong Kong, China; 5 Stanley Ho Centre for Emerging Infectious Diseases, The Chinese University of Hong Kong, Hong Kong, China

## Abstract

**Importance:**

Seroprevalence studies inform the extent of infection and assist evaluation of mitigation strategies for the COVID-19 pandemic.

**Objective:**

To estimate the prevalence of unidentified SARS-CoV-2 infection in the general population of Hong Kong.

**Design, Setting, and Participants:**

A prospective cross-sectional study was conducted in Hong Kong after each major wave of the COVID-19 pandemic (April 21 to July 7, 2020; September 29 to November 23, 2020; and January 15 to April 18, 2021). Adults (age ≥18 years) who had not been diagnosed with COVID-19 were recruited during each period, and their sociodemographic information, symptoms, travel, contact, quarantine, and COVID-19 testing history were collected.

**Main Outcomes and Measures:**

The main outcome was prevalence of SARS-CoV-2 infection. SARS-CoV-2 IgG antibodies were detected by an enzyme-linked immunosorbent assay based on spike (S1/S2) protein, followed by confirmation with a commercial electrochemiluminescence immunoassay based on the receptor binding domain of spike protein.

**Results:**

The study enrolled 4198 participants (2539 [60%] female; median age, 50 years [IQR, 25 years]), including 903 (22%), 1046 (25%), and 2249 (53%) during April 21 to July 7, 2020; during September 29 to November 23, 2020; and during January 15 to April 18, 2021, respectively. The numbers of participants aged 18 to 39 years, 40 to 59 years, and 60 years or older were 1328 (32%), 1645 (39%), and 1225 (29%), respectively. Among the participants, 2444 (58%) stayed in Hong Kong since November 2019 and 2094 (50%) had negative SARS-CoV-2 RNA test results. Only 170 (4%) reported ever having contact with individuals with confirmed cases, and 5% had been isolated or quarantined. Most (2803 [67%]) did not recall any illnesses, whereas 737 (18%), 212 (5%), and 385 (9%) had experienced respiratory symptoms, gastrointestinal symptoms, or both, respectively, before testing. Six participants were confirmed to be positive for anti-SARS-CoV-2 IgG; the adjusted prevalence of unidentified infection was 0.15% (95% CI, 0.06%-0.32%). Extrapolating these findings to the whole population, there were fewer than 1.9 unidentified infections for every recorded confirmed case. The overall prevalence of SARS-CoV-2 infection in Hong Kong before the roll out of vaccination was less than 0.45%.

**Conclusions and Relevance:**

In this cross-sectional study of participants from the general public in Hong Kong, the prevalence of unidentified SARS-CoV-2 infection was low after 3 major waves of the pandemic, suggesting the success of the pandemic mitigation by stringent isolation and quarantine policies even without complete city lockdown. More than 99.5% of the general population of Hong Kong remain naive to SARS-CoV-2, highlighting the urgent need to achieve high vaccine coverage.

## Key Points

**Question:**

What was the prevalence of unidentified SARS-CoV-2 infection in April 2021 after 3 major pandemic waves in Hong Kong, a city without complete lockdown?

**Findings:**

In this cross-sectional study of 4198 participants of the general public in Hong Kong, 6 were identified as positive for anti-SARS-CoV-2 IgG after 3 major waves of COVID-19. The adjusted prevalence of unidentified infection was 0.15%, with fewer than 1.9 unidentified infections for every recorded case.

**Meaning:**

The findings suggest that stringent isolation and quarantine policies even without complete city lockdown are successful in minimizing SARS-CoV transmission.

## Teaser

This cross-sectional study estimates the prevalence of unidentified SARS-CoV-2 infection in the general population of Hong Kong after 3 major pandemic waves.

## Introduction

The COVID-19 pandemic has induced a substantial global health burden.^1,2^ The diagnosis of COVID-19 is confirmed via detection of SARS-CoV-2 RNA by real-time reverse transcription polymerase chain reaction among individuals with an exposure history or indicative clinical features.^3^ However, since the infection can be asymptomatic or involve minimal symptoms and laboratory tests may not be available, a substantial proportion of SARS-CoV-2 infection could be missed.^4^ Underestimation of the true extent of infection at the population level could bias the evaluation of public health policies.^5^

Seroprevalence studies not only inform the extent of an infection, but also play a crucial role in assessing the effects of pandemic mitigation strategies.^6,7,8^ At present, most of the available seroprevalence studies were from hotspots in Europe, America, and mainland China, whereas data from cities with lower attack rates are limited.^5^ Hong Kong is an urbanized metropolitan city ranking the first for population density in the world.^9^ Its close proximity to mainland China, the first epicenter of COVID-19, together with its extensive international traffic make it susceptible to importation of COVID-19 cases and subsequent local transmission.^10^ We examined the prevalence of SARS-CoV-2 IgG after 3 major waves of COVD-19 in Hong Kong, the world city of Asia.^11^

## Methods

### Study Design and Setting

This prospective cross-sectional study was conducted to assess the seroprevalence of SARS-CoV-2 in Hong Kong. A recruitment session was scheduled after each major wave of COVID-19 in Hong Kong. In total, 3 recruitment sessions were conducted from April 21 to July 7, 2020; from September 29 to November 23, 2020; and from January 15 to April 18, 2021, after each major wave. Adults 18 years or older were invited through various public channels, including posters, e-mails, and social media. Individuals with COVID-19 confirmed in Hong Kong or elsewhere were excluded. All individuals were tested once only. Those who had participated in an earlier round of recruitment were excluded to avoid repeated testing. Eligible registrants were stratified according to age, sex, and region of residence and then arranged consecutively for blood sample obtained according to their date of registration. The recruitment started once the COVID-19 wave was under control and was stopped when there was a substantial upsurge of local cases. Registrants were asked to fill in an online survey to record their sociodemographic and medical information; history of travel, contact, quarantine, and COVID-19 testing; and presence of clinical symptoms anytime throughout the pandemic. Written informed consent was obtained from each participant. The study was approved by the Joint Chinese University of Hong Kong–New Territories East Cluster Clinical Research Ethics Committee. This study followed the Strengthening the Reporting of Observational Studies in Epidemiology (STROBE) reporting guideline.^12^

Hong Kong practiced stringent containment measures, including compulsory isolation of confirmed cases and quarantine of all close contacts, but without complete city lockdown. The internationally recognized stringency index developed by the University of Oxford was used as an objective indicator of the intensity of government containment measures implemented during the study period.^13^ This composite measure is based on 9 response indicators including school closures, workplace closures, and travel bans, rescaled to a value from 0 to 100 (100 indicates strictest). The stringency index is for comparative purposes across countries but not meant for assessment of the appropriateness or effectiveness of government response to the pandemic.

### SARS-CoV-2 IgG Detection

An in-house enzyme-linked immunosorbent assay based on recombinant SARS-CoV-2 spike (S) protein consisting of the S1 and S2 subunits (Sino Biological Inc) was used to screen for SARS-CoV-2 IgG antibodies in plasma samples. The assay has been shown to provide a sensitivity of 100% and specificity of 96.9% based on testing of 120 postinfection samples collected between 21 and 125 days after infection and 196 samples collected from healthy individuals before the COVID-19 pandemic. All reactive samples were confirmed by an electrochemiluminescence immunoassay based on the receptor binding domain of S protein, which has been shown to provide a sensitivity of 98.8% and a specificity of 99.9% (Elecsys Anti-SARS-CoV-2 S; Roche Diagnostics GmbH).

### Statistical Analysis

The difference in positive seroprevalence rates observed among recruitment periods was examined by the Fisher exact test with a 2-tailed *P* value of ≤.05 regarded as significant. The 95% CI of the seroprevalence rate was estimated by the Pearson-Klopper method to account for the low rate of IgG positivity. To estimate the observed seroprevalence rate for the whole Hong Kong population, we applied weighted adjustment for the sex and age distribution of the general population in Hong Kong. The estimated demographics of the Hong Kong population by the end of 2020 obtained from the Census and Statistics Department were used.^9^ The daily recorded COVID-19 case numbers were obtained from the Centre for Health Protection of the Government of Hong Kong Special Administrative Region.^14^ EpiInfo, version 7.2 (Centers for Disease Control and Prevention) was used for assessing proportions. R, version 4.1.1 (R Project for Statistical Computing) was used for 95% CI estimation. Excel 2019 (Microsoft Corp) was used for weighted adjustment for sex and age distribution.

## Results

All together, 4198 participants were enrolled in the seroprevalence study, of whom 2539 (60%) were women. Their age ranged from 18 to 88 years (median age, 50 years [IQR, 25 years) (Table 1). Among the participants, 903 (22%), 1046 (25%), and 2249 (53%) were enrolled during April 21 to July 7, 2020; during September 29 to November 23, 2020; and during January 15 to April 18, 2021, respectively (Figure). The numbers of participants aged 18 to 39 years, 40 to 59 years, and 60 years or older were 1328 (32%), 1645 (39%), and 1225 (29%), respectively. The distributions of participants by sex, age, and residential region were similar to those of the whole Hong Kong population (Table 1). A total of 2444 participants (58%) had not traveled outside Hong Kong since November 2019; 2094 (50%) had received negative SARS-CoV-2 RNA test results, and 170 (4%) had contact with patients with confirmed cases. Most participants (2803 [67%]) did not recall any respiratory or gastrointestinal symptoms between September 2019 and blood sample obtainment for antibody test.

**Table 1.** Characteristics of Study Participants

Characteristic	No. (%)	
	Study participants (n = 4198)	Hong Kong population (n = 6 341 600)^a^
Age, y		
18-39	1328 (32)	2 023 400 (32)
40-59	1645 (39)	2 352 300 (37)
≥60	1225 (29)	1 963 000 (31)
Sex		
Female	2539 (60)	4 065 000 (64)
Male	1659 (40)	2 276 600 (36)
Region of residence		
Hong Kong Island	603 (14)	1 208 000 (16)
Kowloon	1168 (28)	2 276 600 (31)
The New Territories and Islands	2427 (58)	3 911 300 (53)
Travel history since November 2019		
Never	2444 (58)	NA
Once	1062 (26)	NA
Twice	427 (10)	NA
≥3 times	265 (6)	NA
History of contact with patients with COVID-19	170 (4)	NA
Was isolated or quarantined	207 (5)	NA
Previous COVID-19 test with negative results^b^	2094 (50)	NA
Symptoms between November 2019 and blood sample obtainment		
None	2803 (67)	NA
Respiratory symptoms with or without fever	737 (18)	NA
Gastrointestinal symptoms with or without fever	212 (5)	NA
Both respiratory and gastrointestinal symptoms with or without fever	385 (9)	NA
Fever only	61 (1)	NA

Abbreviation: NA, not applicable.

^a^ Hong Kong population data are from the Census and Statistics Department, the Government of the Hong Kong Special Administrative Region.^9^

^b^ Six registrants who had SARS-CoV-2 infection confirmed in Hong Kong or overseas were excluded.

**Figure.**
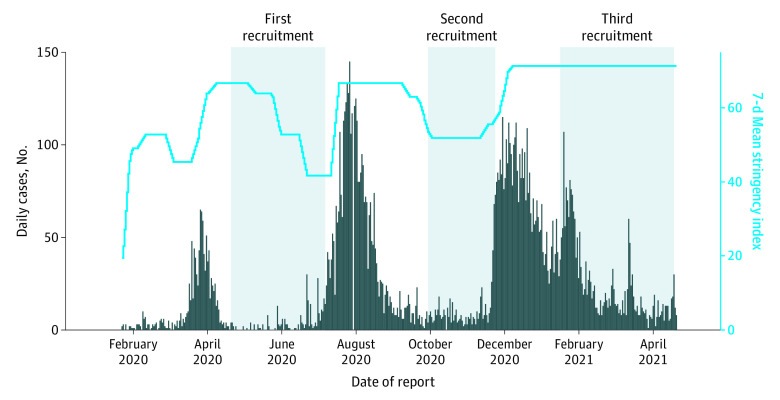
Daily Confirmed COVID-19 Case Numbers, Containment Stringency Index, and Recruitment Period The daily recorded COVID-19 case numbers (vertical bars) were obtained from the Centre for Health Protection of the Government of Hong Kong Special Administrative Region.^14^ The 7-day moving mean containment stringency index (horizontal line) was obtained from Our World in Data.^13^ The stringency index is for comparative purposes across countries but not meant for assessment of the appropriateness or effectiveness of government response to the pandemic. The mean (SD) stringency index in Hong Kong across its pandemic waves was high (62.3 [10.2]) compared with the worldwide mean index (18.0; SD not available).

Overall, 6 participants were confirmed to be positive for anti-SARS-CoV-2 IgG. Two participants with positive results were from the first recruitment, 1 from the second recruitment, and 3 from the third recruitment, which corresponded to positivity rates of 0.22% (2 of 903), 0.10% (1 of 1046), and 0.13% (3 of 2249), respectively, with no significant difference in the positivity rates among the 3 recruitment periods (*P* = .74 by Fisher exact test). The overall observed IgG positivity rate was 6 of 4198 individuals (0.14%; 95% CI, 0.05%-0.31%). All but 1 participant with a positive result had traveled overseas, quarantined, and was tested had negative COVID-19 test results (Table 2).

**Table 2.** Characteristics of 6 Individuals With SARS-CoV-2 IgG Antibodies Detected

Patient No./sex/age, y	Travel history	Contact history	Isolated or quarantined	Previous COVID-19 RNA test	Symptoms
1/F/30s	Short trip to the UK and France in early 2020	Family member had a confirmed case	Home quarantine and isolation camp	Results of multiple deep throat saliva tests at airport and quarantine camp were negative	Fever and chills for a few days while in France
2/M/20s	Studied in the UK; back to Hong Kong for long vacations	Social gathering with a person with a confirmed case for a few hours	Home quarantine	Result of a deep throat saliva test was negative	Frequent cough and occasional sneezing
3/M/20s	Short trips in Taiwan, the UK, and Budapest in late 2019 and early 2020	No	Home quarantine	Result of a deep throat saliva test was negative	Multiple episodes of mild cough and sore throat
4/F/50s	No	No	No	No	No
5/M/40s	Short trip to Ireland in late 2020	No	Hotel quarantine	Results of multiple nasal swab tests were negative	Fever and sore throat for a few days while staying in Ireland
6/M/20s	Studied in the UK; back to Hong Kong for long vacations	No	Hotel quarantine	Result of a deep throat saliva test was negative	Sore throat, loss of taste and smell for a few days while staying in the UK; cold, nausea, and vomiting in February 2021 while in Hong Kong

To estimate the number of unidentified SARS-CoV-2 infections, we applied weighted adjustment according to the sex and age distribution of the general adult population in Hong Kong. The adjusted seroprevalence rate of 0.15% (95% CI, 0.06%-0.32%) was then estimated for the whole adult (age ≥20 years) population of 6 341 600. Based on this rate, we estimated that 9512 (95% CI, 3805-20 293) adults in Hong Kong could be positive for SARS-CoV-2 IgG in addition to the official record of 10 729 confirmed adult cases as of April 18, 2021, the last day of study enrollment.^14^

To estimate the number of unidentified SARS-CoV-2 infections in children, referenced the proportion of recorded confirmed cases among children 17 years or younger as of April 18, 2021, which was 8.2% (955 of 11 684), and assumed that the adult-to-children ratio for unidentified infections was the same as recorded confirmed cases. With this, we estimated that there were 850 (95% CI, 340-1813) unidentified SARS-CoV-2–infected children in Hong Kong.

Therefore, we estimated that the overall number of unidentified infections in Hong Kong was between 4145 and 22 106, with fewer than 1.9 (22 106 divided by 11 684) unidentified infections per every recorded confirmed SARS-CoV-2 infection in Hong Kong. The overall prevalence of SARS-CoV-2 infection before the roll out of vaccination was estimated to be less than 0.45% (upper 95% CI of unidentified infections estimated from the current study [22 106] plus the recorded confirmed cases [11 684] divided by the whole population in Hong Kong [7 474 200]).

Hong Kong experienced 1 initial minor wave and 3 subsequent major waves of COVID-19 before the roll out of vaccination program in early 2021. After the upsurge of cases in each wave, the containment measures were escalated swiftly to a high level, reaching a stringency index of more than 60 (Figure). The mean (SD) stringency index in Hong Kong during the pandemic waves was high (62.3 [10.2]) compared with the world mean of 18.0 (SD not available).^13^

## Discussion

This study examined the prevalence of hidden SARS-CoV-2 infections in the general population that were not identified by the official case findings and reporting system. Our findings represent the prevaccination era in Hong Kong, where the vaccination program was rolled out on February 26, 2021, with a slow uptake. As at the end of the current study on April 18, 2021, only 5% of the population had received 2 doses of COVID-19 vaccine.^15^ We estimated a low adjusted prevalence (0.15%) of unidentified infection compared with the prevalence reported elsewhere.^5^ Our findings suggest that the stringent policies on pandemic mitigation, in particular the compulsory isolation of patients with confirmed cases and quarantine of all close contacts, were successful even without complete city lockdown.^10^ Most of the individuals with unidentified cases revealed in this study had received SARS-CoV-2 RNA testing, but all results were negative. Although these subjects might not have been tested at the right time, the possibility of false-negative results should not be neglected. Of note, the deep throat saliva sample, which was widely used in Hong Kong, has been shown to carry a false-positive rate of up to 31%.^16^

### Limitations

This study has limitations. The study population could have been biased because more health-conscious individuals may have preferred to participate. We could have underestimated the prevalence of infection because those who had been infected a long time ago or with asymptomatic or mild infections may not have retained detectable antibodies. For instance, it has been shown that 28% of health care personnel who had positive SARS-CoV-2 antibody test results seroreverted to below the threshold of positivity approximately 60 days later,^17^ and 40% of asymptomatically infected individuals became seronegative in the early convalescent phase.^18^ Furthermore, we were not able to differentiate whether the infections were acquired overseas or locally.

## Conclusions

In this cross-sectional study of participants from the general public in Hong Kong, the prevalence of unidentified SARS-CoV-2 infection was low after 3 major waves of the pandemic, suggesting the success of the pandemic mitigation by stringent isolation and quarantine policies even without complete city lockdown. An important message from our study is that more than 99.5% of the general population in Hong Kong are naive to SARS-CoV-2. Thus, there is an urgent need to achieve high vaccination coverage to gain individual protection as well as herd immunity to resume normal daily and economic activities.
